# Addressing the range anxiety of battery electric vehicles with charging en route

**DOI:** 10.1038/s41598-022-08942-2

**Published:** 2022-04-04

**Authors:** Prabuddha Chakraborty, Robert Parker, Tamzidul Hoque, Jonathan Cruz, Lili Du, Shuo Wang, Swarup Bhunia

**Affiliations:** 1 Department of Electrical and Computer Engineering, University of Florida, Gainesville, FL USA; 2 Department of Electrical Engineering and Computer Science, University of Kansas, Lawrence, KS USA; 3 Department of Civil and Coastal Engineering, University of Florida, Gainesville, FL USA

**Keywords:** Electrical and electronic engineering, Environmental impact

## Abstract

Battery electric vehicles (BEVs) have emerged as a promising alternative to traditional internal combustion engine (ICE) vehicles due to benefits in improved fuel economy, lower operating cost, and reduced emission. BEVs use electric motors rather than fossil fuels for propulsion and typically store electric energy in lithium-ion cells. With rising concerns over fossil fuel depletion and the impact of ICE vehicles on the climate, electric mobility is widely considered as the future of sustainable transportation. BEVs promise to drastically reduce greenhouse gas emissions as a result of the transportation sector. However, mass adoption of BEVs faces major barriers due to consumer worries over several important battery-related issues, such as limited range, long charging time, lack of charging stations, and high initial cost. Existing solutions to overcome these barriers, such as building more charging stations, increasing battery capacity, and stationary vehicle-to-vehicle (V2V) charging, often suffer from prohibitive investment costs, incompatibility to existing BEVs, or long travel delays. In this paper, we propose Peer-to-Peer Car Charging (P2C2), a scalable approach for charging BEVs that alleviates the need for elaborate charging infrastructure. The central idea is to enable BEVs to share charge among each other while in motion through coordination with a cloud-based control system. To re-vitalize a BEV fleet, which is continuously in motion, we introduce Mobile Charging Stations (MoCS), which are high-battery-capacity vehicles used to replenish the overall charge in a vehicle network. Unlike existing V2V charging solutions, the charge sharing in P2C2 takes place while the BEVs are in-motion, which aims at minimizing travel time loss. To reduce BEV-to-BEV contact time without increasing manufacturing costs, we propose to use multiple batteries of varying sizes and charge transfer rates. The faster but smaller batteries are used for charge transfer between vehicles, while the slower but larger ones are used for prolonged charge storage. We have designed the overall P2C2 framework and formalized the decision-making process of the cloud-based control system. We have evaluated the effectiveness of P2C2 using a well-characterized simulation platform and observed dramatic improvement in BEV mobility. Additionally, through statistical analysis, we show that a significant reduction in carbon emission is also possible if MoCS can be powered by renewable energy sources.

## Introduction

According to the United States (US) Environmental Protection Agency (EPA), transportation takes the largest share (29%) of all economic sectors in terms of greenhouse gas (GHG) emission^1^. Lightweight surface vehicles, such as cars and trucks, are major contributors to GHG emissions in transportation due to their dependence on fossil fuel, with a typical passenger vehicle emitting nearly 4.6 metric tons of carbon dioxide per year. Battery Electric Vehicles (BEVs) are introducing a paradigm shift that promises a dramatic reduction in GHG emissions. The total annual emission from a BEV is less than one-third of a comparable gasoline vehicle on average across the US^2^. While BEVs have zero tailpipe or direct emissions, the total annual emission of a BEV largely depends on the electric energy sources used for charging the battery^2^. The annual emission of BEVs can effectively be made near-zero by using renewable energy sources for battery charging.

**Figure 1:**
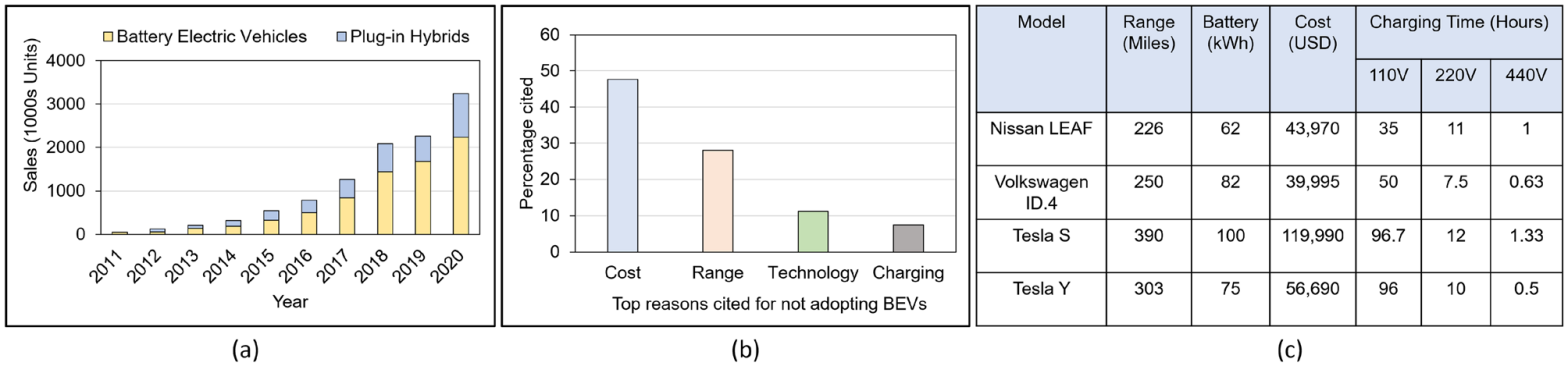
Description of figures from left to right. (**a**) Growth of global BEV sales. (**b**) Problems preventing BEV growth. (**c**) Single charge range and battery charging time of high-end BEVs.

Besides promising a greener alternative to gasoline vehicles, BEVs also provide several other attractive features, such as lower operating costs and lower maintenance. Furthermore, several major car manufacturers, such as Tesla, Nissan, BMW, Mercedes-Benz, and Volkswagen, are making their EV models both appealing and increasingly affordable. As seen in Fig. 1, the sales of BEVs are on the rise. However, there are several key consumer concerns holding BEVs from becoming mainstream and preventing mass adoption. These concerns include battery life, battery range, limited access to charging stations, and high initial cost (mostly attributed to high battery capacity)^3^. Inefficient charging cycles or complete discharge reduces a battery’s life, making it imprudent to travel the full range provided by the battery without periodic recharging^4^. Even though major cities have charging stations, the amount is still unable to support a large BEV population. Charging stations in remote regions are few and far between. Most of the existing charging stations are Level-2 (220 V) which require long waiting periods to charge a vehicle. Level-3 charging stations or DC fast chargers (DCFC) (440 V) are a faster alternative; however, they are limited and very expensive to build^5^. With these concerns in mind, researchers have looked into several alternative solutions. Andwari et al. surveyed innovations in BEV battery technologies^3^, but concluded that the battery range and charging time remains the most critical barrier. Novel solutions like charging via solar-powered roads are not applicable across the geography^6^. V2V stationary charge sharing solutions have also been proposed, but most of these solutions require designated aggregators or charge sharing hubs^7,8^. Additionally, such solutions require the vehicles to be parked during the charge transaction process leading to a significant loss in BEV road time. Wireless charging schemes such as the one proposed by Kosmanos, D.et al.^9^, are hard to implement (cost & feasibility) and will probably be ineffective (wireless charge transfer efficiency is 40–60% at best^10^).

**Figure 2:**
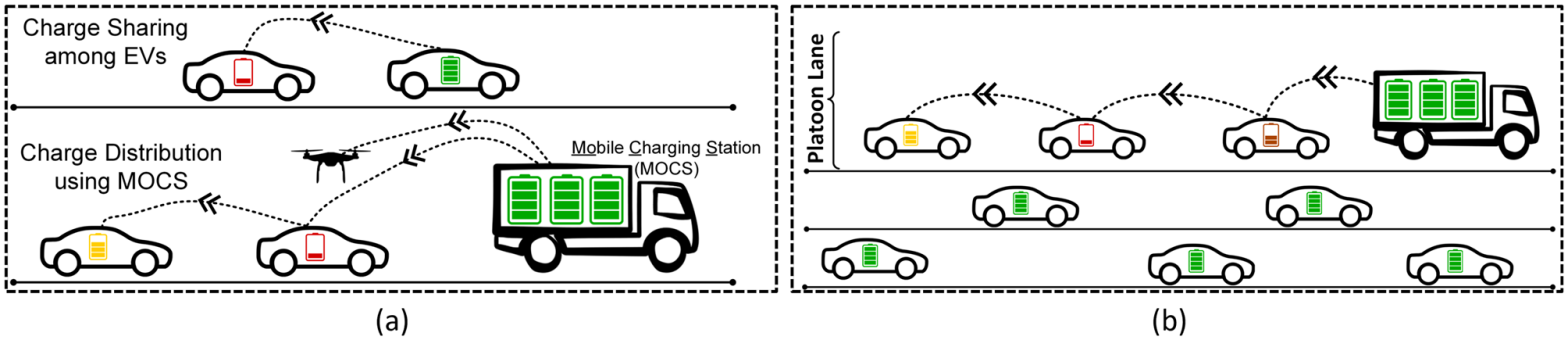
Description of figures from left to right. (**a**) P2C2 enabled charge sharing among BEVs and MoCS-based charge distribution for charging on-the-go^11^. (**b**) A MoCS leader escorting/recharging a BEV platoon.

In this paper, we propose a scalable peer-to-peer on-the-go vehicle charging solution that is both low cost and easy to implement with minimal changes to the BEVs. As shown in Fig. 2, vehicles will share charge and sustain each other to reach their respective destinations. A cloud-based control system determines charge providers/receivers and guides the BEVs to carry out the charge transfer operations while in motion. With this scheme in place, the total charge in the BEV network will eventually spread out across all the BEVs. However, even in a very dynamic system with BEVs entering and leaving the network, we observe through simulation the total charge of the network will slowly deplete. To revitalize the BEV network, we introduce **Mo**bile **C**harging **S**tations (MoCS) that can provide a source of external charge for the BEVs. MoCS recharge nearby BEVs on the go (through platooning) while the BEVs themselves share this charge among each other over a period of time. Reducing the time two vehicles remain connected during charging (contact time) is crucial for convenience and road safety. To address this concern, we explore a multi-level (ML) battery architecture that utilizes a set of fast and slow charging batteries. The battery with a fast charge transfer rate is used to provide/receive charge, while the slow battery powers the BEV. Depending on the situation, the fast-charging battery pumps charge into or out of the slow battery while not in contact with another BEV.

The P2C2 framework consists of (1) the BEV network, (2) the MoCS network, (3) the MoCS depots, and (4) the control system. We formalize the communication and decision-making process of this entire framework to enable efficient BEV-to-BEV pairing, MoCS insertion, BEV/MoCS routing, and multi-level battery state transition. We have designed an evaluation platform through augmenting a popular traffic simulation framework called SUMO^12^ and verified the efficacy of our proposed P2C2 scheme. We have designed a separate evaluation engine for determining the best multi-level battery architecture for a given scenario. We demonstrate that our system is fast, scalable, and efficient in dealing with battery-related problems present in BEVs. We believe that through a combination of battery capacity reduction (possible with P2C2) and solar-powered MoCS, the carbon footprint of BEVs can be further reduced. We present our statistical analysis on this aspect and demonstrate a dramatic reduction in greenhouse gas emissions.

In particular, we make the following major contributions:

1. We introduce a novel solution to address the BEV charging issue by proposing an on-the-go peer-to-peer charge sharing scheme. We formalize a complete framework that enables BEVs to share charge on the go guided by a cloud-based control system.
2. We introduce the concept of mobile charging stations that seamlessly fit into our framework. These mobile charging stations are deployed in charge-deprived regions to boost the overall network charge level.
3. We introduce the idea of multi-level battery architecture, which can help reduce vehicle-to-vehicle contact time during on-the-go charge sharing.
4. We formalize the decision-making process of the control system and propose an algorithm for efficient charge transaction scheduling, optimal MoCS insertion, and optimal rerouting.
5. We quantitatively analyze the effectiveness of our solution using the simulation frameworks that we have developed. Through statistical analysis, we project the effective greenhouse gas emission reduction possible through a P2C2 framework.

## Related works and motivation

In this section, we shall look at different issues preventing BEVs from being widely adopted. We will also analyze some of the proposed solutions and qualitatively compare them to P2C2.

### Impact of battery-related issues on BEV adoption

BEVs have been around since 1823, but despite substantial corporate and government effort, it is still not a viable transport solution for the masses. Several battery-related concerns such as limited range, battery cost, and lack of charging stations have deterred consumers from allowing BEVs to become mainstream.

#### Limited range and lack of charging stations

Long distance driving with a BEV can be difficult due to limited battery range. Detour to reach a charging station, availability of an open charging slot, and charge-up time are the main sources of frustration. Lithium-ion batteries remain expensive to build and greenhouse gas emission from battery manufacturing is becoming a bigger issue^3^. In Fig. 1 we show the range, charging time, and cost of different high-end electric car models. The values reported are for the 2021 Nissan LEAF SL Plus, the 2021 Volkswagon ID.4, the 2021 Tesla S (Tri-Motor All-wheel Drive Plaid), and the 2021 Tesla Y (Performance Dual-Motor All-Wheel Drive). These are approximate values based on an internet survey but they show a clear trend. High-end BEVs such as Tesla Model S and Model Y suffer from high charging times. Most of the charging stations are in urban areas, and most rural areas lack even 110 V charging stations making universal BEV adoption challenging. DCFC (Level-3) stations are scarce and building more is financially challenging^5^.

#### Battery life and high initial purchase cost

The life of a Lithium-ion (Li-ion) battery degrades faster if it is subject to complete discharge or inefficient charging cycles. Li-ion batteries are widely used in BEVs^13^. Hence, completely draining the BEV battery may be undesirable to the car owners. Hence, if the user chooses to avoid accelerated battery ageing, then it virtually decreases the BEV’s range. Also, BEVs are generally more expensive than their traditional ICE vehicle counterparts due to high battery manufacturing cost.

### Existing solutions to address charging issues

Issues relating to the battery and charging appears to be the core hurdle preventing a full-scale adoption of BEVs. Next, we shall discuss some of the proposed existing solutions aimed at countering battery related issues in BEVs. Table 1 provides a comparison among existing solutions and P2C2 (proposed).

**Table 1:** Comparison between P2C2 and other BEV sustainability solutions.

Solution	Cost	Deployability	Mobility	Charge transfer mode	EV-to-EV charging	On-the-go	Multi level battery
Dense CS	Very high	Hard	Low	Physical	No	No	No
Improved Battery	Very high	Moderate	–	–	–	–	No
Charging from Road^6^	Very high	Hard	High	Physical	No	Yes	No
V2V (Hub)^8,14–17^	High	Moderate	Low	Physical	Yes	No	No
V2V (Direct)^7,18^	Moderate	Easy	Low	Physical	Yes	No	No
Charging Trucks/Robots^19,20^	Moderate	Moderate	Low	Physical	No	No	No
Dynamic Charging^9^	High	Very hard	Low	Wireless	No	Yes	No
Battery Swapping^21,22^	Very high	Hard	Moderate	–	–	No	No
**P2C2 (proposed)**	**Moderate**	**Moderate**	**High**	**Physical/drone**	**Yes**	**Yes**	**Yes**

#### More charging stations, higher battery capacity and battery swapping

Building a large number of very high speed (Level-3) charging stations in close proximity can alleviate range anxiety. However, dense and uniformly placed Level-3 stations are not financially feasible. Additionally, even a Level-3 charging station is not fast enough to allow a seamless long drive experience; hence, even faster charger stations are required. Furthermore, the local power grids must be re-designed to handle the huge load due to these fast BEV charging stations^23^. Increasing the BEV battery size can enable long-distance travel and in turn reduce range anxiety. However, this solution is expensive and not scalable^3^. Manufacturing larger batteries will also increase greenhouse gas emissions making BEVs less attractive. It also does not solve the core battery re-charging problems.

Several research and industry efforts are also being made towards developing battery swapping techniques^21,22^. However, such battery swapping stations are very expensive to build and a large number of such stations will be required to support a big BEV fleet. Directly accessing the BEV battery (mostly located at the base of the BEV to lower the center of gravity) is also challenging and will require major changes to the core BEV architecture.

#### Stationary V2V charge sharing

Several solutions have been proposed around the idea of BEV-to-BEV charge sharing at designated hubs. A hub can be an aggregator or a charging station. In works such as^8,22^, the BEVs parked at a hub share charge among each other and the grid to optimize overall charging efficiency. The aggregator can also allow direct V2V charge sharing bypassing the grid^15–17^. Such a hub will be less expensive to build than a charging station because no grid connectivity is required.

The idea of trucks distributing charge to regions lacking charging stations has been proposed in^19,24,25^. The trucks initially receive charge at a depot and then travel to a designated spot in which this charge can be distributed via stationary V2V charging. Additionally, to counter the lack of BEV charging ports in parking lots, the concept of a robot-like charging entity has been proposed that can move around the parking lot and serve multiple BEVs^20^.

However, relying on designated hubs such as aggregators and charging stations to share charge is both expensive and inconvenient due to significant infrastructure requirements. Hence in works such as^7,18^, the authors experiment with V2V charge sharing without the availability of any designated hubs. The game theory based solution in^7^ achieved improved charge sharing efficiency in comparison to other techniques. Yet, for all of these solutions, the BEVs must be parked at equipped parking lots and remain stationary during the entire charging process.

#### Charging from the road and dynamic charging

Charging BEVs from the road can be an effective solution, but it may not be the most efficient. A road in Normandy, France, was fitted with solar panels to generate electricity in 2018. It produced only a total of 80,000 kWh in that year and about 40,000 kWh by the end of July 2019^6^. The lack of efficiency was due to (1) Normandy’s climate (average 44 days of sunshine), (2) damaged solar panels, and (3) obstructions from leaves. Converting every major roadways in the world into electric/solar roads is a big financial undertaking, rendering this solution practically infeasible.

A wireless charging solution was proposed by Kosmanos, D.et al.^9^ which involves charging BEVs from a Bus or Truck. State-of-the-art wireless charge transfer techniques have efficiencies of about 40–60%. A coil of 340 cm or 11.15 feet in diameter has a maximum 60% power transfer efficiency while transmitting across 170 cm or 2.2 feet^10^. Such a small distance is extremely unsafe for on-the-go charging in most traffic scenarios and building/hosting such huge coils on both the receiver and the transmitter can be challenging.

### Why on-the-go charging?

Refueling of an ICE vehicle is both fast and easy to the point that it is not even a concern, no matter how long a trip is. Similarly, if re-charging a BEV can be achieved without long wait time, meticulous planning, and lengthy detours, then ICE vehicle owners may get enticed to make the switch to BEVs. Solutions such as increasing battery size and building faster charging stations only serve as band-aids to the inherent BEV battery-related problems. Although V2V charging schemes can somewhat mitigate the lack of charging stations, it does not eliminate the need to remain stationary while charging and endure long travel time loss. The only functional on-the-go charging solution, solar road charging, although intriguing, is not financially feasible.

We hypothesize that if BEV-to-BEV charge sharing can be done on-the-go (while in motion), then it can (1) eliminate re-charging wait time, (2) increase battery life by avoiding inefficient charging cycles, (3) eliminate range anxiety by reducing reliance on charging stations, (4) reduce BEV cost by eliminating the need to have big batteries, and (5) reduce greenhouse gas emission if MoCS are powered via renewable sources. Based on this hypothesis, we design our peer-to-peer on-the-go charging system called P2C2.

## Peer-to-peer charging methodology

Our proposed framework, P2C2, enables BEVs to carry out charge transactions between them while on-the-go. In this paper, we build on the fundamental principles presented in^11^ and enhance the potency of the methodology by introducing the concept of multi-level battery architecture that acts as a charge-caching mechanism for decreasing BEV-to-BEV contact time. We validate this concept by designing a multi-level battery simulation setup and performing a comprehensive study on the effectiveness of P2C2 when utilizing multi-level battery architectures. Additionally, we discuss different mechanical/electrical aspects of on-the-go charge sharing and investigate possibilities for greenhouse gas emission reduction through P2C2.

### System overview

**Figure 3:**
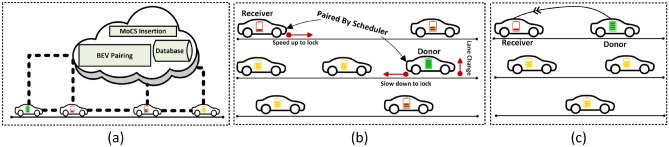
(**a**) In a P2C2 framework, BEVs and MoCS interact with each other and a control system for information/instruction sharing. The control system located in the cloud facilitates BEV-to-BEV charge sharing and optimal MoCS insertion. (**b**) The paired BEVs are being guided by the control system to move closer and share charge. (**c**) Paired BEVs speed lock and share charge on-the-go^11^.

**Figure 4:**
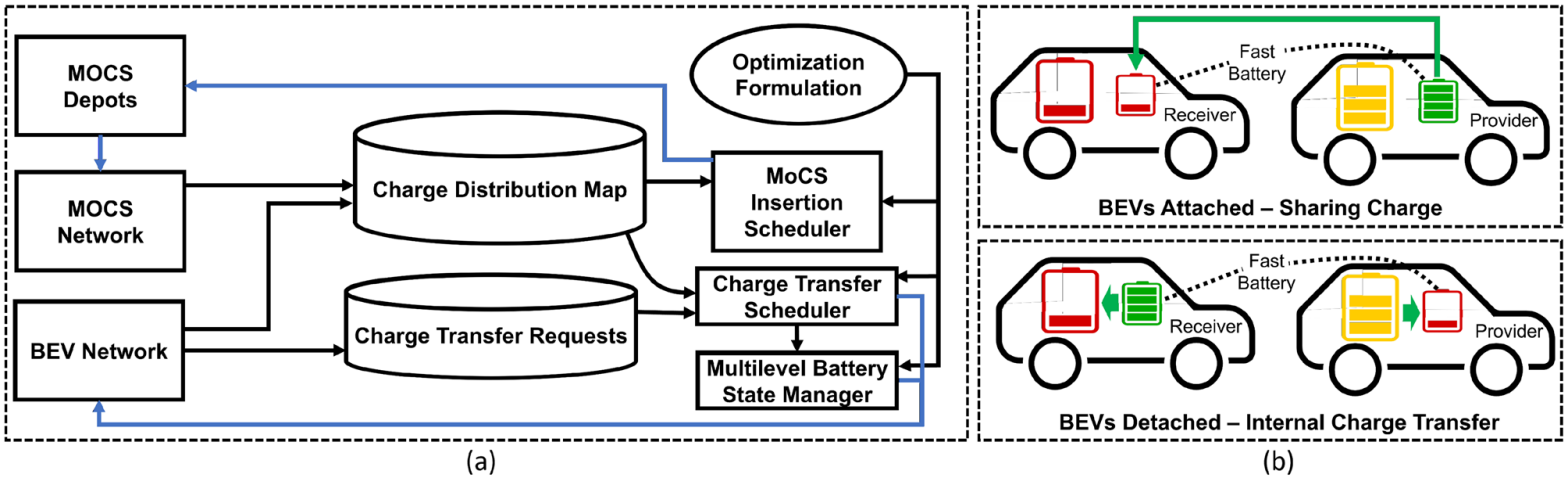
Description of figures from left to right. (**a**) The system-level view of the P2C2 framework shows the data and control flow between different entities. (**b**) With a two-level battery architecture, the fast (but smaller) battery can be used for BEV-to-BEV charge transfer and once detached, the smaller battery can recharge the slower-main battery.

The P2C2 framework consists of a BEV network, a MoCS network, MoCS depots, and a cloud-based control system. The BEVs/MoCS interact with each other and the control system, as shown in Fig. 3a for sharing traffic and battery information. This information is used to update a charge distribution map while charging requests from BEVs are gathered in the charge transfer request database for subsequent processing. In Fig. 4a (left sub-figure), we present an information/control flow system view of the P2C2 framework. Based on an optimization algorithm, the control system (1) chooses/instructs some BEVs to share charge with other BEVs, (2) reroutes specific BEVs to bring charge providers and receivers together, (3) speed lock BEVs to allow seamless charge sharing on-the-go, and (4) detaches charge provider/receiver pairs as required, for overall network charge optimization. An example BEV-to-BEV synchronization for charge sharing is shown in Fig. 3b, c.

Sharing charge between BEVs will eventually distribute the total charge in the network among all the entities. But we observe through simulation that without an outside-the-network charge source or sufficient BEV inflow, the network will experience a slow overall charge decay. To avoid this problem, we introduce **Mo**bile **C**harging **S**tations (MoCS) which brings in a high volume of charge into the network periodically at key locations. The BEVs themselves are then responsible for a more granular distribution of the charge deposited by MoCS, based on the control system’s instructions. MoCS are strategically inserted by the control system based on the charge distribution map and optimization goals. Figure 2 shows a MoCS charging a set of BEVs in a lane to form a charge sharing platoon. MoCS hubs host the MoCS and charge them up after they return from charge distribution trips.

### Multi-level battery architecture

The charge transfer rate between BEV-to-BEV and MoCS-to-BEV is the main bottleneck for any system such as P2C2. Reducing contact time between vehicles while on-the-road is important in terms of system efficiency and safety. Manufacturers can increase the battery charge transfer rate for the entire battery to reduce contact time but incur higher manufacturing costs. To tackle this problem, we draw inspiration from memory caching mechanisms used in modern computer systems where a hierarchy of memory units of varying speed/size are used to optimize performance and cost^26^. Similarly, we propose the use of a set of batteries with varying charge transfer rates and sizes to optimize contact-time and cost. Only the faster batteries are responsible for transferring charge between the BEVs while they are connected, leading to a reduced BEV-to-BEV contact time. The primary difference between fast-charging batteries and regular batteries is that the fast-charging batteries can take higher charging power than the regular batteries without degrading the battery performance and lifetime. Therefore, these batteries can be charged quickly. On the other hand, fast-charging batteries typically have a higher cost than regular batteries. As a result, the proposed multi-level battery technique is a good balance of charging speed and cost. In Fig. 4b (two sub-figures on the right), a two level battery system is depicted where the fast-small batteries are being used to share charge between the BEVs. Once the receiver fast battery is fully charged, or the provider fast battery is almost completely drained, the BEVs detach. While detached, the BEV in *provider* state will pump more charge into its fast battery, and the BEV in *receiver state* will transfer charge from the fast battery to the slow/bigger battery. This allows the receiver BEV to get ready for receiving charge and also prepares the provider BEV for serving subsequent charge transfer requests. This concept can also be used in MoCS for fast charge delivery with reduced contact time.

Offloading the charging process of the slow/bigger battery to the detached state reduces contact time while increasing system safety and traffic efficiency. The multi level battery architecture can reduce contact time with little effect on the manufacturing cost. Through extensive simulations, we provide evidence for this claim in “Effectiveness of ML battery architecture” section.

### Network optimization and scheduling algorithm

At the core of the P2C2 framework resides the control system, which is responsible for optimal BEV-to-BEV pairing, MoCS insertion, and multilevel battery state switching. All these tasks can be envisioned as an optimization problem, and in Table 2, we list a set of core parameters and variables for this optimization task. Based on the application scenario, we first capture the requirements using an optimization formulation as shown in Eqn. (1). This formulation is then periodically referred to by different entities in the system to make optimal decisions (see Fig. 4a, left sub-figure).

1 $$\begin{aligned} \begin{aligned} \begin{array}{ll} \vec {x} = <x_1, x_2, \ldots , x_n> &{}\quad {\text {A set of variables which can be controlled by the system.}}\\ \min /\max f_m(\vec {x}),\; m = 1, 2, \ldots , M &{}\quad {\text {A set of optimization goals crafted from different parameters.}}\\ weights = [w_1, \ldots , w_M] &{}\quad {\text {We add a weight to every goal to emphasize its importance.}}\\ {\text {s.t.}} \; g_t(\vec {x}) \ge 0,\; t= 1,2, \ldots , T &{}\quad {\text {A set of constraints crafted from different parameters.}}\\ h_q(\vec {x}) = 0, \;q= 1,2, \ldots , Q &{}\quad {\text {A set of constraints crafted from different parameters.}}\\ x_i^{LB} \le x_i \le x_i^{UB},\; i= 1,2, \ldots , n &{}\quad {\text {A set of bounds for all variables.}}\\ \end{array} \end{aligned} \end{aligned}$$

**Table 2:** Proposed core parameters and variables for the P2C2 framework.

Parameters		Variables	
Charge loss ($p_1$)	Travel time loss ($p_2$)	MoCS insertion decisions ($x_1$)	BEV-to-BEV pairing decisions ($x_2$)
Charging station halts ($p_3$)	Battery life degradation ($p_4$)	MoCS pathway ($x_3$)	BEV rerouting decisions ($x_4$)
Network charge level ($p_5$)	MoCS overhead ($p_6$)	Multilevel battery architecture ($x_5$)	Multilevel battery state switching ($x_6$)
BEV-to-BEV contact time ($p_7$)	Traffic flow ($p_8$)	BEV-to-BEV detachment decisions ($x_7$)	Platooning decisions ($x_8$)

In Algorithm 1, we depict the decision making mechanism of the system utilizing the optimization formulation. The control system takes in the charge distribution map ($Charge\_Dist\_Map$), the list of BEVs requesting for a provider BEV ($Charge\_Transfer\_Req$), and the scenario specific optimization settings ($Optimization\_Formulation$) as inputs. Between lines 5-12, we show the major steps for determining (1) the ideal provider BEV for the $i^{th}$ request ($Charge\_Transfer\_Req[i]$) and (2) the associated multi level battery state transitions. For that, we first identify a set of $Local\_BEVs$ around the receiver BEV and select the most suitable candidate ($Prov\_BEV$) from the set. The optimization formulation guides this decision process. Based on the selected provider-receiver BEV pair, any multi level battery state transition requirements are determined ($Multi\_Level\_Battery\_State\_Transition\_Ins$). The pairing instructions and the multi level battery state transition instructions are collected in the lists (1) $BEV\_Pairing\_Instructions$ and (2) $Multi\_Level\_Battery\_State\_Instructions$ respectively. In the second phase of Algorithm 1, we show the major decision steps involving MoCS insertions. For each charge deprived region ($Charge\_Deprived\_Regions[i]$), we determine where to insert the MoCS ($MoCS\_Pathway$) and (2) the amount of MoCS to insert ($MoCS\_Count$). These decisions are also influenced by the optimization settings ($Optimization\_Formulation$) and the instructions are collected in $MoCS\_Depot\_Instructions$.

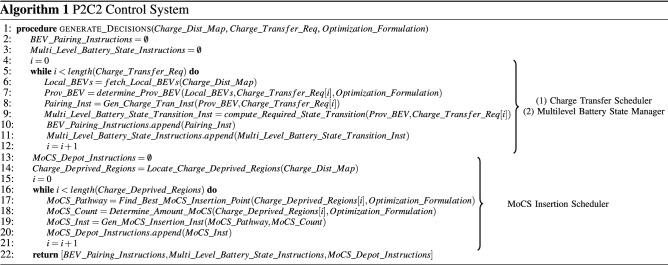



## P2C2: traffic simulation results

For evaluating the P2C2 scheme, we have developed a simulation framework and performed a set of quantitative analysis. We have modified the SUMO (Simulation of Urban Mobility)^12^ traffic simulator to support peer-to-peer BEV charging on-the-go, MoCS, and MoCS hubs. We couple the enhanced SUMO framework with our P2C2 control system algorithm to create a complete simulation environment. We next test P2C2 in different traffic settings and parameters.

### Simulation setup and fundamental observations

The P2C2 control system communicates with SUMO periodically to gather traffic information and send instructions. We use a 240 km highway to test our method. We run each simulation instance for 5 hours in real-time. Each BEV weighs 2109 kg with a battery capacity of 75 kWh. Unless otherwise mentioned, the BEVs and MoCS enter the simulation with a full charge. The weight of each MoCS is 11793 kgs which is the gross vehicle weight rating for a class 6 truck^27^. Each MoCS carries 850 kWh charge for distribution and its operation. In our analysis, we observe the effect of other parameters such as (1) MoCS-to-BEV charge transfer rate, (2) amount of MoCS in the network, and (3) battery capacity reduction of the BEVs in later sections.

We test most of our observations on three different traffic scenarios. The internal parameters defining each of these scenarios are as follows:

1. **Light traffic** Initially, 500 BEVs are inserted with a new BEV entering the simulation every 4 seconds. A total of 5000 BEVs will be inserted over 5 hours.
2. **Medium traffic** Initial traffic of 1000 BEVs with a new BEV entering the simulation every 3 seconds. A total of 7000 BEVs will be inserted over 5 hours.
3. **High traffic** Initially, 2000 BEVs are inserted with a new BEV entering the simulation every 2 seconds. A total of 11000 BEVs will be inserted over 5 hours.

We use a charging rate of 1kW/min for simulation based on a realistic BEV-to-BEV charging estimate provided in^28^. We consider a BEV to be halted when its charge reaches zero. All charge transfer is carried out with 95% efficiency (i.e., 5% loss during transfer). For these experiments, we use the following optimization formulation (see Eq. (2)) to capture the system requirements based on the parameters and variables presented in Table 2. Here *MO* indicates the limit on the amount of MoCS that can be inserted in the system.

2 $$\begin{aligned} &\vec {x} = \langle x_1, x_2, x_3, x_4, x_7, x_8 \rangle \\&\min f_{p1}(\vec {x}), \min f_{p2}(\vec {x}), \min f_{p3}(\vec {x}), \min f_{p4}(\vec {x}) \\&\max f_{p5}(\vec {x}), \min f_{p6}(\vec {x}), \min f_{p7}(\vec {x}), \max f_{p8}(\vec {x}) \\&{\text {s.t.}} \; g_{p6}(\vec {x}) \le MO \end{aligned}$$

**Figure 5:**
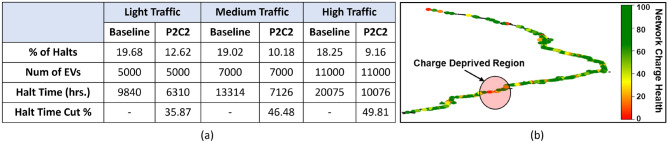
Description of figures from left to right. (**a**) The percentage of halt induced charging time reduction in different traffic scenarios. (**b**) The charge distribution map maintained by the cloud application at a particular time instance^11^.

**Figure 6:**
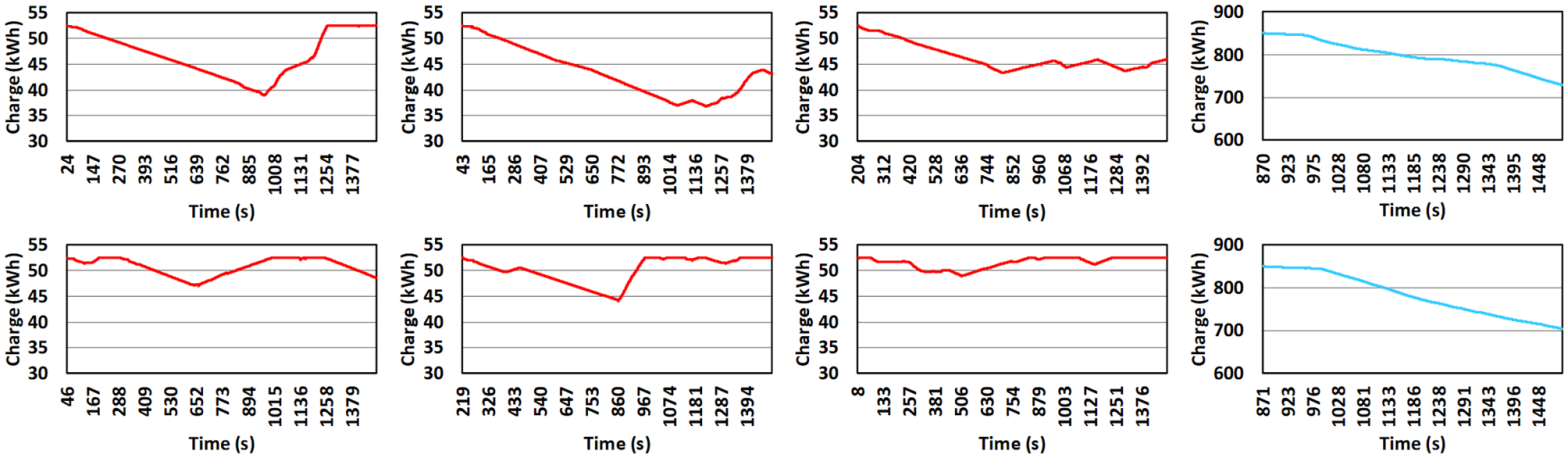
Change of battery charge levels over time for sampled BEVs(red) and MoCS(blue) in the network^11^.

Figure 5b (right sub-figure) illustrates a sample overall charge distribution in the highway. Each point on the plot indicates the average charge of vehicles in the region. In the charge distribution map shown (Fig. 5b), we can observe a potential charge deprived region. The scheduler will then assess the current status of the network to determine the number of MoCS to be inserted at the charge deprived region. In Fig. 6, we can see the battery charge trends for 6 sampled BEVs (red) on the left and 2 sampled MoCS (blue) on the right from the network. The BEVs generally experience an initial drop in the battery charge before they are assigned another BEV as a provider. After that point, most of the BEVs maintain a steady battery level and continue to move perpetually. The purpose of MoCS is to deposit a huge amount of charge in the network quickly; hence, they constantly lose charge, as can be seen from the blue plots.

### Effect of MoCS charge transfer rate on amount of halts

**Figure 7:**
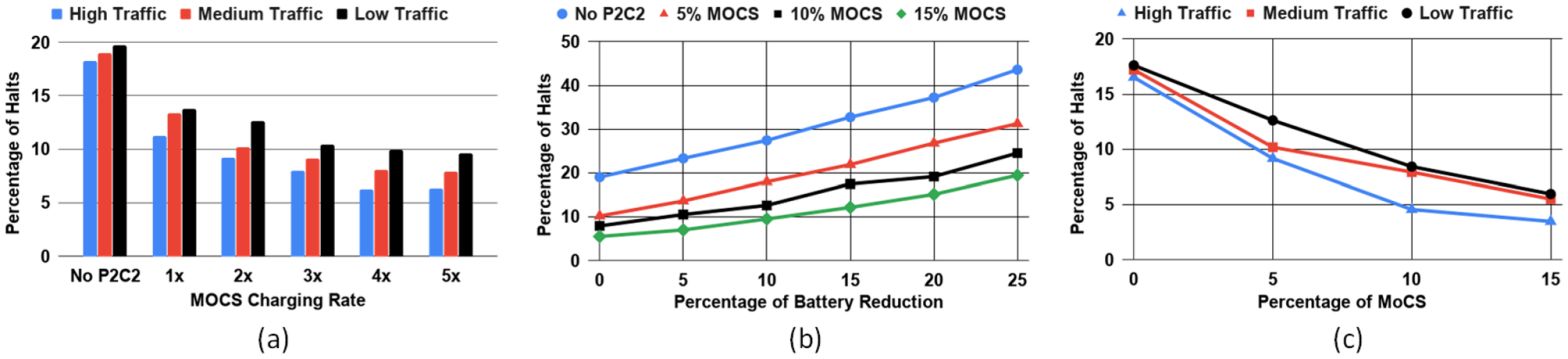
(**a**) The percentage of BEV halts reduces as the MoCS-to-BEV charge transfer rate increases. (**b**) The percentage of halt increases as we decrease the battery capacity. The halt percentage is less with more MoCS in the system. (**c**) The percentage of BEV halts reduces as the limit on the percentage of MoCS in the network is increased^11^.

We observe the effect of different MoCS-to-BEV charge transfer rates on the percentage of BEV halts. $1\times$ charge rate is 1 kWh per minute based on^28^. Note that we only change the charge transfer rate between a MoCS and a BEV. The BEV-to-BEV charge transfer rate remains 1 kWh per minute throughout the experiment. In Fig. 7a, we observe that the percentage of halts for all the three traffic scenarios decreases as we increase the MoCS charge transfer rate. If fast charge transfer batteries can be used in the BEVs/MoCS, then the effectiveness of P2C2 will be increased. P2C2 charging scheme appears to be more effective in denser traffic scenarios. As can be seen in Fig. 7a, the percentage of halts for high traffic is the least. With more BEVs in the network, less amount of rerouting is needed, and a BEV with a critical battery state can be quickly assigned to a provider BEV which is close by. Active MoCS at any time is limited to 5% of the entire BEV/MoCS fleet size.

### Battery capacity reduction and MoCS tradeoff

High capacity batteries in BEVs lead to increased weight and cost. In Fig. 7b, we observe the effect of reducing the battery capacity of the BEVs on the percentage of halts for the medium-traffic scenario. We see the percentage of halts increases as the battery capacity is reduced. There is a trade-off possibility between the amount of MoCS and the battery capacity of BEVs. If the amount of MoCS inserted is within 15% of the total BEVs in the network, we can reduce the battery capacity of all BEVs by 24.4% and still achieve the same amount of halts compared to not using the P2C2 scheme. Therefore, we can reduce the battery capacities of all BEVs just by having more MoCS in the system. In future work, we will look into incorporating cost in this trade-off. For this experiment, we set the MoCS-to-BEV charge transfer rate to $2\times$ (2 kWh per minute), and BEV-to-BEV charging rate to $1\times$ (1 kWh per minute).

### Effect of number of MoCS on percentage of halts

To observe the effect of the number of MoCS in the network on the percentage of BEV halts, we set the MoCS-to-BEV charge transfer rate to $2\times$ (2 kWh per minute), and BEV-to-BEV charging rate to $1\times$ (1 kWh per minute) and vary the limit on the percentage of MoCS in the network. The percentage of MoCS refers to the maximum allowable MoCS for every 100 BEVs in the network. In Fig. 7c, we observe that as we increase the limit of the percentage of MoCS, the percentage of BEV halts decreases. So a higher quantity of charge influx also helps in halt reduction.

### Charging time reduction analysis

Based on the battery capacity of the BEVs used in the simulation, it should take approximately 10 hours to fully charge on the NEMA 14-50 plugs through a 240 V outlet^29^. By multiplying the average charging time for each halt with the total number of halts, we obtain the total charging time for all traffic scenarios. As shown in the table in Fig. 5a, the total time spent on stationary charging reduces significantly due to the charge sharing scheme proposed. The % of reduction for P2C2 is calculated compared to the required charging time results for no P2C2 (without). We use a BEV-to-BEV charging rate of $1\times$, a MoCS-to-BEV charging rate of $2\times$, and a 5% MoCS amount limit for obtaining these results.

## Effectiveness of ML battery architecture

So far, we have provided evidence (in a traffic simulation setting) that the P2C2 scheme with a single monolithic/traditional battery has several benefits. Next, we will look at the benefits of using a multi-level battery architecture coupled with peer-to-peer on-the-go charging.

### Mobility

For a BEV travelling on a road network, factors such as vehicle-to-vehicle contact time, number of halts, and length of halts affect its mobility and the mobility of the entire traffic. We quantitatively define mobility using Eq. (3). Here *T* is the time spent by a BEV in contact with another BEV/MoCS while charge sharing using the P2C2 scheme, *n* is the number of halts and *H* is the halt penalty. The halt penalty *H* is computed as shown in Eq. (4). Here *C* is the battery charging time. The factor 2 is multiplied because *C* is the contact time at the charging station and *C* is also the travel time loss.

3 $$\begin{aligned} Mobility = (T + nH)^{-1} \end{aligned}$$

4 $$\begin{aligned} H = 2\times C \end{aligned}$$

To simulate and capture the mobility values of different P2C2 and Non-P2C2 systems, we implement a set of simulators which we shall describe next. After that, we will analyze the mobility values for different battery architectures and systems.

### Mobility simulators

To evaluate the effectiveness of multi-level (ML) battery architectures (described in “Multi-level battery architecture” section), we have designed a set of simulators described using Algorithms 2, 3, and 4.

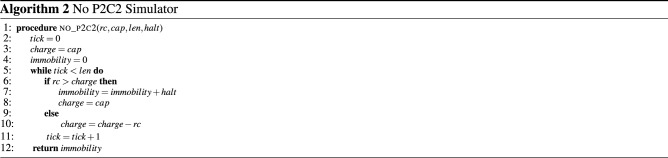

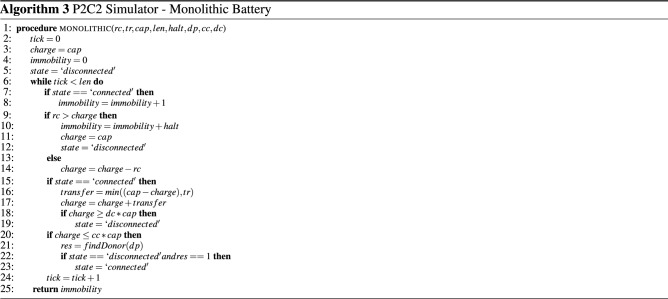

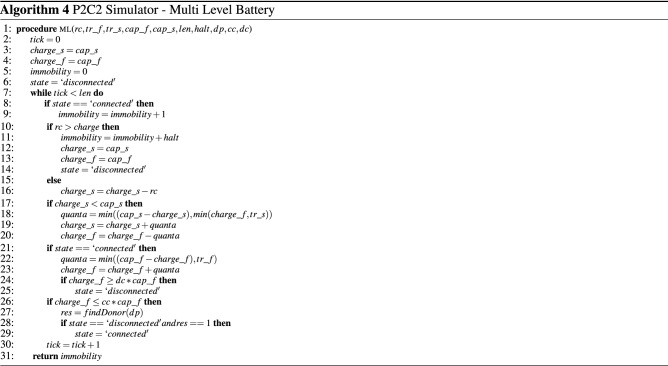







The mobility simulator for the system with no P2C2 is described using Algorithm 2. Here *rc* is the running cost of the BEV in terms of kWh/min, *cap* is the battery capacity of the BEV, *len* is the simulation length in minutes, *halt* is the halt penalty of the BEV computed as shown in Eq. (4). *rc* is deducted from the *charge* while the BEV is in motion (line 10). When the BEV runs out of battery, a halt penalty is added to the immobility variable, and the BEV battery is set to full again (lines 6-8).

The mobility simulator for the system with P2C2 and a monolithic BEV battery is described using Algorithm 3. In addition to the parameters used for Algorithm 2, we introduce *tr* which is the charge transfer rate of the BEV battery in terms of kWh/min, *dp* which is the charge provider finding probability, *cc* which is the battery capacity cutoff below which the BEV will start searching for a provider, and *dc* is the battery threshold above which the BEV remains disconnected from any provider. *rc* is deducted from the *charge* while the BEV is in motion (line 14). When the BEV runs out of battery, a halt penalty is incurred (line 10). While connected to a provider, the BEV battery charges based on the transfer rate *tr* (lines 15–17). Once charged beyond $dc\times cap$, the BEV detaches from its provider. If the battery is below $cc\times cap$, the BEV starts searching for a provider BEV (lines 20–23) and the chance of success depends on *dp*.

The mobility simulator for the system with P2C2 and multi-level BEV battery is described using Algorithm 4. We have designed the simulator for a two-level BEV battery architecture. The battery pack consists of a fast battery and a slow battery. In addition to some of the parameters used for Algorithm 3, we introduce $tr\_f$ which is the charge transfer rate of the fast battery, $tr\_s$ which is the charge transfer rate of the slow battery, $cap\_s$ which is the capacity of the slow battery, and $cap\_f$ is the capacity of the fast battery. *rc* is deducted from the $charge\_s$ (slow battery charge) while the BEV is in motion (line 16). When the BEV runs out of battery, a halt penalty is incurred (line 11). While connected to a provider, the BEV’s fast battery charges up based on its charge transfer rate $tr\_f$ (lines 21–25). If the slow battery is not full, then the fast battery recharges the slow battery (lines 17–20).

The inverse of the immobility values computed from the simulators is the corresponding mobility metric values.

### Contact time reduction using multi-level battery

**Figure 8:**
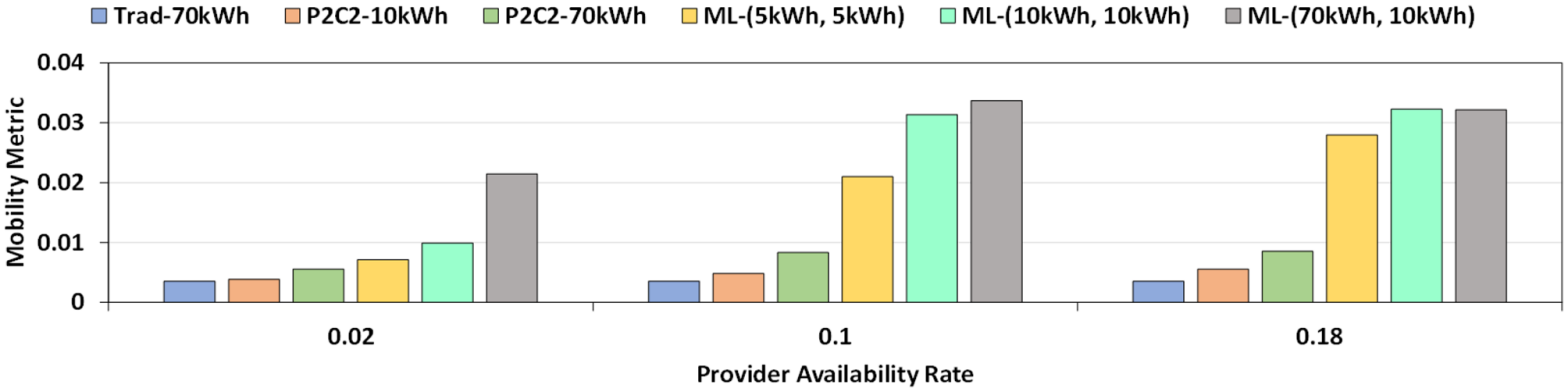
Mobility of vehicular networks for different battery sizes and provider availability rate. Trad refers to a system without P2C2 and a monolithic battery. P2C2 refers to a system with a monolithic battery and peer-to-peer on-the-go charging. ML refers to a system with both peer-to-peer on-the-go charging and BEVs with multi-level battery. We observe that multi-level battery systems offer higher mobility at lower combined battery sizes.

**Figure 9:**
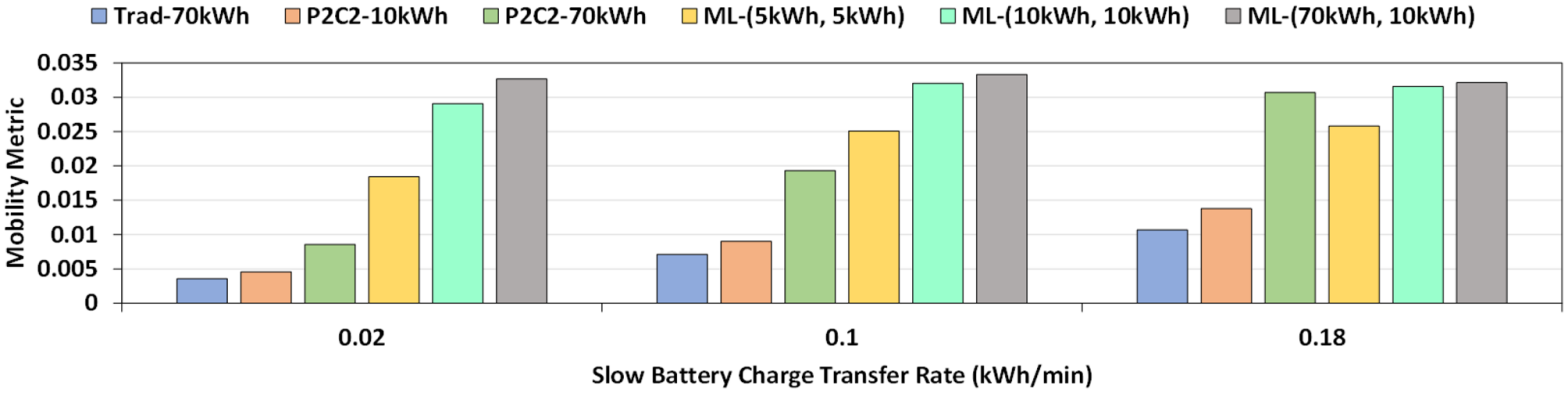
Mobility of BEV networks for different battery sizes and battery charge transfer rate. Even at a higher charge transfer rate, Trad/No-P2C2 system suffers from low mobility while ML battery systems perform well even at low rates.

We observe that the contact time between BEVs can be significantly reduced and mobility of the system can be enhanced if multi-level batteries are used instead of a single monolithic battery as can be seen in Figs. 8 and  9. All the experiment results are averaged over 10 independent runs. *rc* is fixed at 0.25 kWh/min, *len* is 600, *cc* is 0.2 and *dc* is set to 0.8. Additionally for the results in Fig. 8, $tr\_s$ is 1 kWh/min and $tr\_f$ is set to 5 kWh/min. For the results in Fig. 9, *dp* is 0.1 and $tr\_f$ is set to 5 kWh/min. A multi-level battery system not only improves mobility but also can help reduce the battery size of the BEV. For example, in the case of ML-(10 kWh, 10 kWh), a fast battery of size 10 kWh and a slow battery of 10 kWh can outperform a BEV with a slow 70 kWh battery in any setting. Reducing battery size will lead to a reduction in ${\text {CO}}_2$ emission during manufacturing, cut down cost and decrease car weight.

## Discussion

We have presented different direct benefits of P2C2 using SUMO and the proposed simulation frameworks. In this section, we shall look into a few additional benefits of P2C2 and explore future research directions.

### Potential for carbon emission reduction

BEVs are not entirely emission-free, even after they are manufactured. If grid electricity is used for charging a BEV, then technically, the BEV is responsible for the ${\text {CO}}_2$ emission due to the electricity generation for the grid which in most countries, including the USA, are primarily powered by coal, natural gas, and petroleum. Harvesting renewable resources is a cleaner alternative. If BEVs can be completely powered from renewable sources, such as solar, then the carbon footprint from BEVs will drastically decrease. However, it may prove difficult to require all public and private charging stations in crowded cities to harvest energy completely via solar.

**Figure 10:**
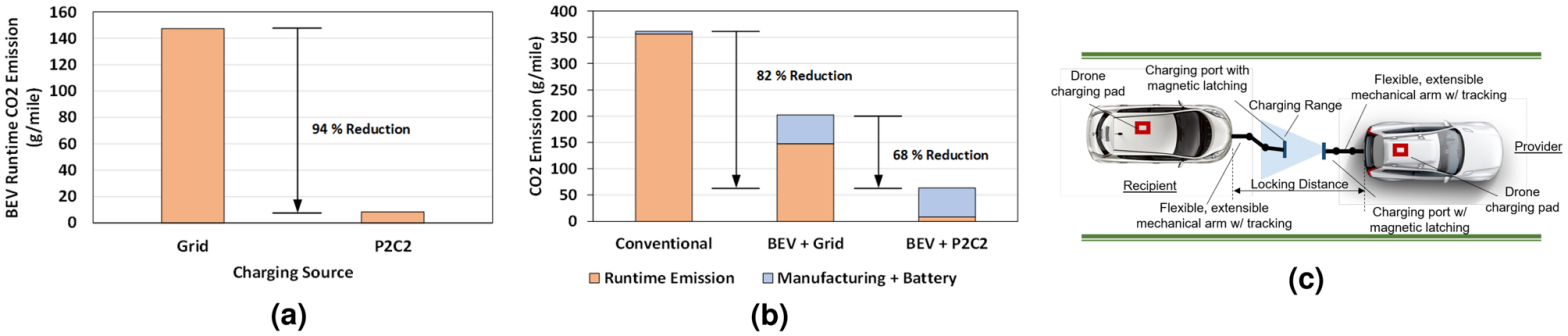
(**a**) Potential carbon footprint reduction during the operation lifecycle of a BEV if MoCS hubs are powered by solar. (**b**) Carbon footprint reduction considering emissions from manufacturing and battery production. (**c**) Proposed peer-to-peer wired charging scheme for moving vehicles.

P2C2 offers an alternative solution by allowing the MoCS Hub to run on solar energy instead. The MoCS hub can be located on the outskirts of cities and on the side of highways where they will have enough open space to construct appropriate solar harvesting facilities. MoCS hubs can be powered by alternate renewable sources as well. The MoCS can then distribute this energy to the BEVs inside dense cities and throughout the whole network removing the burden from the BEV users to reduce their carbon footprints. As seen in Fig. 10a, we estimate a 94% reduction in terms of ${\text {CO}}_2$ emission if BEVs can be sustained by solar-powered MoCS. To obtain this estimate we assume a BEV powertrain efficiency of 4.8 miles/kW^30^, BEV lifetime of 200,000 miles, 707 g ${\text {CO}}_2$ per kWh for energy production for the grid^31^, and 40 g ${\text {CO}}_2$ per kWh for energy production via solar^32^.

To capture the complete picture, in Fig. 10b, we factor in the emissions from BEV manufacturing and battery production. We observe that BEV with P2C2 charging scheme and solar-powered MoCS can potentially lead to a 68 % reduction in ${\text {CO}}_2$ footprint compared to BEV’s running from grid electricity. The reduction is even more when compared to conventional cars. To compute the values we estimate the manufacturing emission of a conventional car to be 5.2g ${\text {CO}}_2$/mile (Kobayashi, 2007)^33^, emission from conventional car to be 356g ${\text {CO}}_2$/mile^34^, and emission for BEV manufacturing (including battery production) to be 55g ${\text {CO}}_2$/mile (for average Tesla BEVs)^30^. For MoCS impact, we first consider the amount of MoCS required. According to several studies such as^35,36^, only about 3–6 % of the cars are used at any time in most of the parts of the world. If we maintain a MoCS team of 15% of the active fleet size, the MoCS team size with respect to the whole BEV population will be only about 0.67%. Therefore, the ${\text {CO}}_2$ impact is negligible given the MoCS battery size is around 10x that of a typical BEV.

### Car-to-car charging mechanism

Transferring the charge from one BEV to another within a limited time while in motion is a challenging task. We propose two instances for charge sharing. For one instance of transferal, we envision a fast, safe, and flexible wired charging connection that is controlled by an autopilot system, a charging port tracking, and a protection system between the BEVs for charge sharing as shown in Fig. 10c. After two BEVs lock speed and are in range for charge sharing, one of the BEVs will extend the charging arm that will attach with the other BEV or MoCS. The two charging pads will be securely latched for energy sharing. In the second instance, the charge transfer can also be performed using drones. In this case, the drone can land on the charging pad on a moving vehicle, and the charging can be finished by wired means. The drone can just fly away after the charging is completed and land back on the provider’s pad.

**Figure 11:**
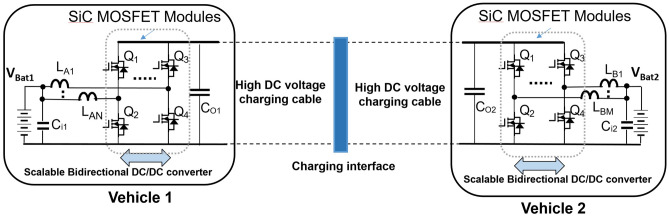
Proposed interleaved, high power, scalable, and bidirectional on-board Buck/Boost DC/DC converters for peer-to-peer BEV charging.

Figure 11, illustrates an interleaved, Buck/Boost, high voltage DC/DC converter charging circuit with SiC MOSFET for scalable power and bidirectional power flow control which can be utilized for BEV-to-BEV charge sharing. The charging circuit works at Boost mode when the battery voltage from the charging donor vehicle is increased to high voltage along the cables to reduce the charging current, therefore reduce the power loss due to high charging power on the cables and devices. The high voltage from the cable is reduced to battery voltage at Buck mode on the charging receiver vehicle for battery charging. The phase-shifted interleaving structure can help improve power scalability, reduce current ripples and reduce electromagnetic interference.

**Figure 12:**
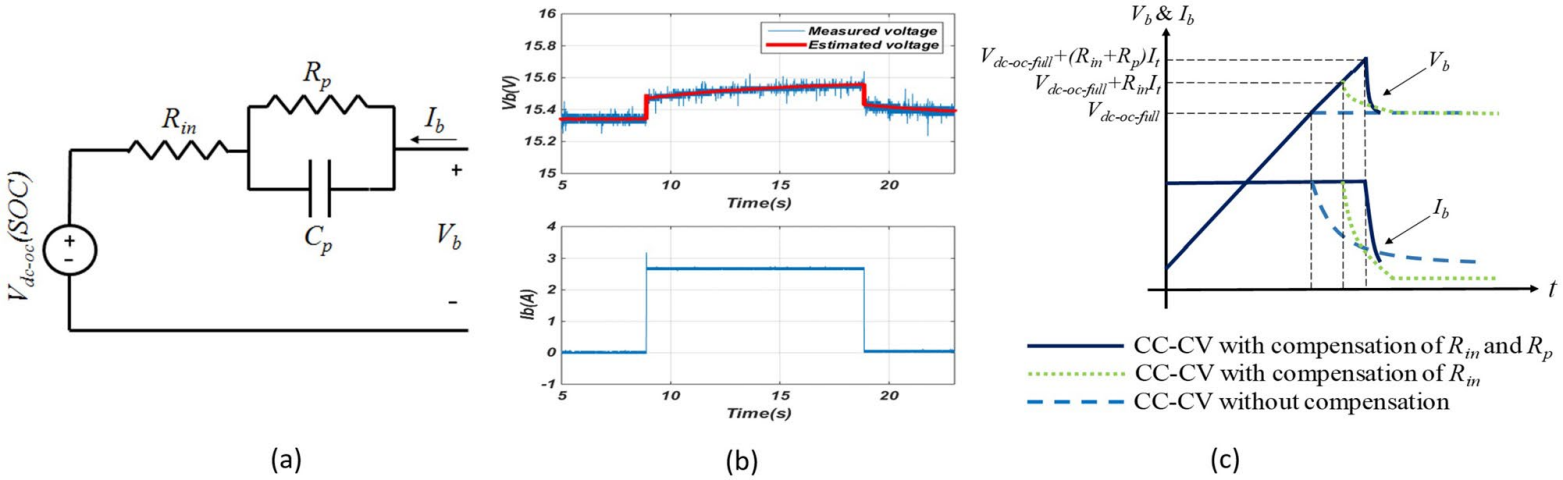
Proposed strategy for fast charging of Lithium-Ion batteries: (**a**) Lithium-ion battery model, (**b**) single-pulse charging current technique to derive the internal resistance and polarization parameters of lithium-ion battery, and (**c**) charging speed comparison between conventional CC–CV fast charging technique and the proposed CC–CV fast charging with internal resistance and polarization parameter compensation.

Additionally, we envision the P2C2 framework incorporating fast charge sharing techniques to reduce contact time as shown in Fig. 12. A novel internal resistance and polarization parameter detection technique will be used to detect the polarization parameters of the batteries as shown in Fig. 12a and b using a single-pulse technique. Based on the detected internal resistance and polarization parameters, the optimal charging scheme will be programmed to achieve fast charging as shown in Fig. 12c. The charging speed is more than 20% faster than the state-of-art charging schemes. At the same time, the short circuit protection will be initiated if the internal resistance is smaller than the threshold resistance. Active cancellation and passive shielding techniques will be implemented to reduce the radiated EMI along the charging cables so the whole charging system can meet safety and electromagnetic compliance requirements.

Our fast charging scheme is developed based on a real-time battery model in Fig. 12a, b and the voltage drop on internal resistance $R_{in}$ and $R_p$ can be compensated during the constant current (CC) charging stage which has a fast-charging speed^37^. As a result, in Fig. 12c, the CC charging stage can last longer without battery degradation issues, and the constant voltage (CV) charging stage (slow charging speed for battery safety) is significantly reduced. Because of this, the total charging time can be significantly reduced. Furthermore, since the charging is managed based on the battery’s state of health, state of charge, and the open-circuit voltage predicted in real-time, compared with the conventional CC–CV charging scheme, the battery’s lifetime can be increased with the proposed fast charging scheme.

### P2C2 and platooning

Multiple vehicles travelling in close proximity to one another for increased fuel efficiency, traffic flow enhancement, safety and other benefits are collectively referred to as a platoon. Hence the act of platoon formation is described as platooning^38^. Platooning is best achieved when a cloud-based control system guides each individual platoon member to maintain a specific distance and position with respect to others^39^. Platooning of trucks is also possible to save energy and increase fuel efficiency^40^. We believe that peer-to-peer charge sharing and platooning fit very comfortably with each other and both these techniques can benefit from the other. Platooning can ensure a safe physical connection between BEVs for charge transfer while the P2C2 framework can ensure optimal platoon formation for charge sharing. Charging a platoon with MoCS is also much easier because the MoCS can easily sustain a series of BEVs if they are all interconnected. If BEVs can be escorted by MoCS for long trips, then the battery capacity of BEVs can be drastically reduced, which in turn can lead to lower manufacturing costs and carbon emissions. Many cruise control technologies, such as ACC, CACC, and connected and autonomous vehicle platooning control enable two vehicles to move fast and safely in a relatively stable and small space^41–51^. These technologies can be used to coordinate the movement of two cars for safely conducting the charging on the move.

## Conclusion

We have presented a framework for charging BEVs on-the-go to address issues such as lack of BEV charging infrastructure, limited range, battery related high BEV cost, and greenhouse gas emissions. P2C2 relies on BEV-to-BEV coordination as well as a cloud-based guidance system for creating a real-time charge distribution map of the network of BEVs and making informed decisions about charge transactions. We incorporate the concept of MoCS—a mobile charging vehicle with a large battery—that can be dispatched to recharge a network of BEVs. We have developed a system and associated algorithms to enable BEV-to-BEV charging. Using a popular traffic simulator, SUMO, we have simulated the P2C2 framework with realistic charging parameters and observed a reduction in the number of halts and battery capacity requirements (thus, leading to reduced cost and weight). We also introduce a cost-effective multi-level battery scheme for efficient battery charge transfer in-motion. We have demonstrated reduced vehicle-to-vehicle contact time and increased vehicle mobility with such a technique. Based on statistical analysis, we observe that P2C2, through solar-powered MoCS can also lead to a dramatic reduction in ${\text {CO}}_2$ emission. Future works will involve extending our solution to heterogeneous networks of battery-operated entities, like drones, and utility robots. We will also investigate real world implementation of this framework in controlled settings using physical prototypes.
